# Effects of dietary inclusion of high concentrations of crude glycerin on meat quality and fatty acid profile of feedlot fed Nellore bulls

**DOI:** 10.1371/journal.pone.0179830

**Published:** 2017-06-23

**Authors:** Eric H. C. B. van Cleef, André P. D'Áurea, Vanessa R. Fávaro, Flavia O. S. van Cleef, Robson S. Barducci, Marco T. C. Almeida, Otávio R. Machado Neto, Jane M. B. Ezequiel

**Affiliations:** 1 São Paulo State University (Unesp), School of Agrarian and Veterinarian Sciences, Jaboticabal, São Paulo, Brazil; 2 São Paulo State University (Unesp), School of Veterinary Medicine and Animal Science, Botucatu, São Paulo, Brazil; University of Illinois, UNITED STATES

## Abstract

Crude glycerin, the main by-product of biodiesel production, can replace dietary energy sources, such as corn. The objective of this study was to evaluate the inclusion of up to 30% of crude glycerin in dry matter (DM) of the total diets, and its effects on meat quality parameters of feedlot Nellore bulls. Thirty animals (227.7 ± 23.8 kg body weight; 18 months old) were housed in individual pens and fed 5 experimental diets, containing 0, 7.5, 15, 22.5 or 30% crude glycerin (DM basis). After 103 d (21 d adaptation) animals were slaughtered and the *Longissimus* muscle was collected. The characteristics assessed were chemical composition, fatty acid profile, cholesterol, shear force, pH, color, water-holding capacity, cooking loss and sensory properties. The increasing inclusion of crude glycerin in the diets did not affect the chemical composition of the *Longissimus* muscle (*P* > 0.10). A quadratic effect was observed when levels of crude glycerin were increased, on the concentration of pentadecanoic, palmitoleic and eicosenoic fatty acids in meat (*P* < 0.05), and on the activity of the delta-9 desaturase 16 and delta-9 desaturase 18 enzymes (*P* < 0.05). The addition of crude glycerin increased the gamma linolenic fatty acid concentration (*P* < 0.01), and altered the monounsaturated fatty acids in *Longissimus* muscle of animals (*P*_*quad*._ < 0.05). Crude glycerin decreased cholesterol content in meat (*P* < 0.05), and promoted higher flavor score and greasy intensity perception of the meat (*P* < 0.01). The inclusion of up to 30% crude glycerin in Nellore cattle bulls`diets (DM basis) improves meat cholesterol and sensory attributes, such as flavor, without affecting significantly the physical traits, the main fatty acid concentrations and the chemical composition.

## Introduction

Nowadays, there is a growing interest in manipulating the carcass quality and fatty acid composition of livestock`s meat, in order to produce meat with higher acceptance by the market, especially in terms of reduced content of saturated fatty acids (SFA), which are directly related to human diseases associated with modern life [1 – 2]. Several studies were recently conducted in order to obtain healthy animal products for human consumption, keeping or increasing nutritional value of meat and not encumbering the animal production systems.

The intensification of livestock production leads to the search for alternatives to reduce the costs of feedstuff, such as the inclusion of by-products to replace conventional feed ingredients. Crude glycerin is a by-product of the biodiesel industry widely used in diets for ruminants, as it has great potential for replacing energetic ingredients, such as corn grain [3 – 4 – 5]. The glycerol (main constituent of crude glycerin) can be converted to glucose by the liver and kidneys to provide energy for cellular metabolism. In ruminants, the glycerol is fermented in the rumen into short chain fatty acids, mainly to propionic and butyric [6]. Recent studies have demonstrated that the inclusion of this by-product has effects on carcass grade [4 – 7], increases the intramuscular fat and oleic acid content [8], decreases myristic, palmitic and stearic acids in *Longissimus* muscle [9], increases the monounsaturated fatty acid (MUFA) content and conjugated linoleic acid content [10], decreases saturated fatty acid and increases unsaturated and odd-chain fatty acid contents [4]. However, in other studies the effects of crude glycerin on carcass and meat traits were neglected [11 – 12 – 13].

Nevertheless, to date there is no study, evaluating high inclusions of crude glycerin in feedlot Nellore cattle diets and its effects on meat quality. Therefore, the objective of this study was to evaluate the inclusion of up to 30% of crude glycerin in DM of the diets, on meat quality parameters of feedlot Nellore bulls.

## Material and methods

### Ethical approval

The Animal Welfare and Ethics Commission from São Paulo State University approved all the procedures involving animals (Protocol Number 010707).

### Animal housing, management and experimental diets

This study was carried out at the Animal Unit of Digestive and Metabolic Studies, and at the Meat Technology Laboratory from São Paulo State University (Unesp), Jaboticabal, São Paulo, Brazil.

Thirty Nellore bulls (227.7 ± 23.8 kg body weight; 18 months old) were individually weighed, tagged, dewormed, supplemented with vitamins A, D, E and K, vaccinated, and housed in individual semi-roofed pens (10 m^2^), equipped with individual feed bunks and waterers. Animals were blocked by initial body weight, and randomly assigned to one of five treatments.

Five diets (Table 1) were formulated with similar concentrations of crude protein (12.2%, DM basis) and metabolizable energy (2.5 Mcal/kg DM), according to recommendations of NRC [14]. Animals received the diets as total mixed rations twice daily (0700 and 1700 h) at a concentrate:roughage ratio of 70:30. Treatments consisted of increasing inclusion of crude glycerin, which mainly replaced dietary corn grain and soybean hulls. Experimental diets were labeled as: G0 = control diets with no crude glycerin addition; G7.5 = with 7.5% crude glycerin in diet DM; G15 = with 15% crude glycerin in diet DM; G22.5 = with 22.5% crude glycerin in diet DM and G30 = with 30% crude glycerin in diet DM.

**Table 1:** Percentage of feed ingredients and nutrient composition of experimental diets.

Item	Treatments^1^				
	G0	G7.5	G15	G22.5	G30
**Ingredients (% DM)**					
Corn silage	30	30	30	30	30
Corn grain	35	25.5	18	12.5	5
Soybean hulls	19.2	18.1	14.6	8.9	5.5
Sunflower meal	14.6	17.8	21.3	24.9	28.4
Crude glycerin	-	7.5	15	22.5	30
Salt (NaCl)	0.5	0.5	0.5	0.5	0.5
Limestone	0.7	0.7	0.6	0.7	0.7
Dicalcium phosphate	-	0.1	0.1	-	-
**Calculated nutrients**					
CP (% DM)	12.2	12.2	12.2	12.2	12.2
ME (Mcal/kg DM)	2.5	2.5	2.5	2.5	2.5
EE (% DM)	2.9	2.6	2.3	2.1	1.8
NDF (% DM)	40.8	40.1	38.1	35	33.1
ADF (% DM)	25.4	25.6	24.7	22.9	22.1
HEM (% DM)	15.4	14.5	13.4	12.1	11
Ca (% DM)	0.6	0.6	0.6	0.6	0.6
P (% DM)	0.3	0.3	0.4	0.3	0.3
**Fatty acids (% FAME)**					
C12:0	0.08	0.09	0.11	0.11	0.14
C14:0	0.14	0.16	0.19	0.18	0.22
C15:0	0.03	0.05	0.05	0.05	0.07
C16:0	12.92	12.83	13.01	12.62	12.74
C16:1	0.14	0.14	0.14	0.13	0.13
C17:0	0.14	0.17	0.18	0.18	0.2
C17:1	0.04	0.05	0.05	0.05	0.05
C18:0	3.00	3.25	3.31	3.44	3.56
C18:1n9	32.51	31.01	29.94	29.82	28.62
C18:1n7	0.93	1.29	1.42	1,42	1,58
C18:2n6	44.97	45.11	45.4	45.95	46.15
C18:3n6	0.03	0.03	0.02	0.03	0.02
C18:3n3	2.98	3.73	4.03	3.87	4.41
C20:0	0.76	0.73	0.71	0.7	0.68
C20:1n9	0.32	0.31	0.3	0.3	0.28
C20:2	0.02	0.02	0.02	0.02	0.02
C20:3n6	0.02	0.02	0.02	0.02	0.02
C22:0	0.44	0.51	0.56	0.6	0.6
C23:0	0.06	0.06	0.07	0.07	0.08
C24:0	0.47	0.44	0.47	0.44	0.43
SFA	18.04	18.29	18.66	18.39	18.72
UFA	81.96	81.71	81.34	80.19	79.7
MUFA	33.94	32.8	31.85	30.3	29.08
PUFA	48.02	48.91	49.49	49.89	50.62

^1^G0 = without crude glycerin, G7.5 = 7.5% crude glycerin in diet DM, G15 = 15% of crude glycerin in diet DM, G22.5 = 22.5% crude glycerin in diet DM, G30 = 30% crude glycerin in diet DM.

The crude glycerin used in the current study was soybean-based and composed of 5% water, 86% glycerol, 6% salts (98% NaCl) and less than 0.01% methanol. This by-product was weighed at the time of feeding, top-dressed on corn silage, and manually mixed at the feed bunks.

Animals were submitted to a 21-d adaptation period to experimental facilities, handling and diets, in which animals received four step-up diets containing increasing levels of concentrate and crude glycerin, and to an 82-d finishing period.

### Slaughter and meat collection

At the end of the feedlot period (d 82) animals were transported to a commercial abattoir located at Barretos, Brazil (100 km away from the research facility), and were submitted to a 16-h solid fast period with free access to water prior to harvesting. After 24-h chill period, samples of *Longissimus* muscle were collected from the left side of the carcass (from 13^th^ rib), cut in four 2.54 cm-thick steaks, vacuum packed into polyethylene bags (water vapor permeability < 10 g/m^2^/24 h at 38°C and oxygen permeability < 40 mL/m^2^/24 h at 25°C), and transported to the Meat Technology Laboratory at Unesp and stored at -20°C.

### Meat centesimal composition

One steak of each animal was thawed at room temperature, cut in small cubes and freeze-dried for 72 h. After the lyophilization process, samples were ground and the centesimal composition was evaluated. Moisture content was determined using a 105°C oven for 16 h [15] (method 967.03); crude protein was estimated using N value from micro-Kjeldahl method [15] (method 920.87), and multiplied by 6.25; ether extract was obtained using the Soxhlet apparatus with petroleum ether as solvent [16] (method 960.39), and mineral fraction was obtained incinerating the samples using a muffle furnace at 600°C [15] (method 942.05).

### Meat and subcutaneous fat color and meat pH

The color parameters of meat and subcutaneous fat were evaluated as described by Houben *et al*. [17], using a colorimeter (Minolta Chroma Meter CR-300, Osaka, Japan) with aperture of 8 mm, illuminant D65, 10° standard observer, open cone. The calorimeter calibration was performed, at room temperature (25°C), before readings using a pure white standard (100% reflection) and a black box (zero reflection). The parameters evaluated were: lightness (L*), redness (a*), and yellowness (b*) which were assessed by the CIE L* a* b* color system [18]. Approximately 30 min before color readings, the muscle`s myoglobin was exposed to oxygen. The color was read at three different points, and the averages were calculated. The device calibration was performed before the readings with white and black standards. The pH was then measured also at three different locations across the surface, using a digital pH-measuring instrument (Testo, model 205).

### Water holding capacity, cooking loss and Warner–Bratzler shear force

The water holding capacity (WHC) was measured submitting approximately 2 g of meat to 10 kg pressure for 5 min. The difference between the meat weight before and after the procedure was used to calculate WHC, expressed as % [19].

For cooking loss (CL) determination, one steak of each animal was thawed at room temperature, weighed, grilled at 200°C pre-heated clamshell grill. When the center of the steak reached 71°C (monitored with a thermometer), the grilling was interrupted and the steaks were reweighed after reaching room temperature. The difference of steaks`weight before and after grilling was considered the CL. The same steaks used for CL determination were used for evaluation of Warner–Bratzler shear force (SF). Round cores (1.27 cm diameter) of meat, free of visible fat and connective tissue, were cut from each steak, parallel to the long axis of the muscle fibers [20], and each core was sheared perpendicularly to the fiber direction using a Warner–Bratzler shear apparatus (G-R Manufacturing Company, Manhattan, KS, USA). The equipment was set to have a crosshead speed of 200 mm/min using a Texture Analyzer TA–XT2i (Stable Micro Systems Ltd., UK). Shear force values were recorded in kgf, and then converted to Newtons (N).

### Meat and subcutaneous fat cholesterol and fatty acid profile

Cholesterol analysis were performed according to Al-Hasani *et al*. [21], involving alcoholic KOH saponification of the samples, extraction of the non-saponifiable fraction with hexane, and injection of concentrated extract into the gas chromatograph (model 14-B, Shimadzu, Kyoto, Japan).

The fatty acids were extracted according to methodology proposed by Bligh *et al*. [22] with some modifications. Approximately 3 g of freeze-dried meat samples (without subcutaneous fat) were transferred to a 125-mL Erlenmeyer, and 10 mL of chloroform, 20 mL of ethanol, and 8 mL of distilled water were added. The reactants were mixed for 30 min in a horizontal shaker (model SL-0031, Solab, Piracicaba, Brazil), and then 10 mL of chloroform and 10 mL of 1.5% sodium sulfate solution were added for another agitation for 2 min. All content was filtered with a quantitative filter paper and transferred to 50-mL Falcon^®^ flasks. After separation, the upper layer was discarded, and 10 mL was transferred to glass beakers previously tarred. The recipient was placed into a 55°C forced air oven, for 24 h, in order to evaporate solvent. After cooling, beakers were reweighed and fat content calculated by difference.

For the trans esterification of triglycerides, approximately 50 mg of lipids were transferred to 15-mL Falcon flasks, and 2 mL of heptane were added. The mixture was agitated until complete fat dissolution, and then 2 mL of KOH (2 mol/L methanol) were added. The new mixture was vigorously agitated for 5 min. After phase separation, 1 mL of upper layer, composed of heptane and fatty acids methyl esters (FAME) was transferred to 1.5-mL microtubes and frozen at -18°C until analyses.

Fatty acid profile analyses were performed using a gas chromatograph (model 14-B, Shimadzu, Kyoto, Japan), along with fused silica capillary column, type Omewax250 (30 m × 0.25 mm × 0.25 μm) Cat. No. 24136-Supelco, with the following analytical conditions and programing: 100°C for 2 min; heating 4°C/min up to 220°C and maintaining this temperature for 25 min; detector temperature of 280°C; injector temperature of 250°C; carrier gas velocity (H_2_) of 1 mL/min; SPLIT 1:100; injection volume of 1 μL; using flame ionization detector. The flux of gases was 23, 50 and 180 kPa, respectively for synthetic air, H_2_ and N_2_. The FA profile of TMR was determined using the same gas chromatograph and procedures. Individual fatty acids were identified by comparison of the retention times with standards (Supelco 37 components FAME Mix, USA).

The Δ9 desaturase (16 and 18) and elongase activities were estimated according to Malau-Aduli *et al*. [23], and the atherogenicity index was estimated according to Ulbricht *et al*. [24]. The equations used were:

Δ9 desaturase 16: 100 [(C16:1cis9)/(C16:1cis9 + C16:0)]

Δ9 desaturase 18: 100 [(C18:1cis9)/(C18:1cis9 + C18:0)]

Elongase: 100 [(C18:0 + C18:1cis9)/(C16:0 + C16:1cis9 + C18:0 + C18:1cis9)]

Atherogenicity: [C12:0 + 4(C14:0) + C16:0]/ΣUFA.

### Sensory analysis

Two 2.54 cm thick steaks were cooked in an electric oven at 175°C, until the geometric center reached 71°C. Cooked steaks were cut in cubes (2 cm^3^), wrapped in aluminum foil and offered to 9 trained panellists, which scored from 1 (minimum acceptance) to 9 (maximum acceptance), according to Meilgaard *et al*. [25], in two sessions (morning and afternoon). Panellists tested 4 samples of each treatment (total of 20 samples), and evaluated the following meat attributes: appearance, odor intensity, flavor intensity, tenderness, juiciness, greasy intensity and overall acceptance.

### Statistical analysis

Data were analyzed as a completely randomized block design using the MIXED procedure of SAS 9.1 (SAS Inst., Inc., Cary, NC). Animals were blocked by initial body weight and each animal was considered an experimental unit. Model effects included treatment (fixed effect) and block (random effect), according to the equation:

Yij=μ+τi+βj+εij,

where:

Yij = observed measurement, μ = overall mean; τi = inclusion level of crude glycerin (i = 0, 7.5, 15, 22.5, 30%); βj = effect of block (j = 1 to 6); and εij = experimental error.

The model for sensory analysis included the fixed effect of treatment and the random effects of panel session (morning or afternoon), panellist, and sample order. Orthogonal contrasts were used to determine the linear and quadratic effects of glycerin, and 0% glycerin × glycerin treatment. Means of treatments were obtained with the LSMEANS option. Significance was declared as *P* ≤ 0.05 and tendency as *P* < 0.10.

## Results

Feeding Nellore feedlot cattle up to 30% crude glycerin (DM of the diet) for 103d did not change performance of the animals (*P* > 0.10, Table 2).

**Table 2:** Dry matter intake and performance of Nellore bulls (n = 30) fed diets containing up to 30% crude glycerin.

Item^2^	Treatments (% crude glycerin)					Contrast, *P*-value^1^			SEM
	G0	G7.5	G15	G22.5	G30	L	Q	0 × Gly	
Initial BW, kg	279.5	280.5	270.5	279.3	278.5	NS^3^	NS	NS	4.46
Final BW, kg	413.9	427.6	423.1	427.3	403.5	NS	NS	NS	15.84
DMI, kg/d	8.96	7.81	8.49	8.75	7.79	NS	NS	NS	0.38
ADG, kg/d	1.54	1.69	1.75	1.7	1.44	NS	NS	NS	0.15
G:F, kg/kg	0.19	0.22	0.21	0.2	0.19	NS	NS	NS	0.02

^1^Linear, Quadratic, Control × glycerin treatments.

^2^DMI = Dry matter intake, ADG = Average daily gain, IBW = Initial body weight, FBW = Final body weight, G:F = Gain to feed. [3]

^3^NS = Not significant.

### Meat centesimal composition

The increasing inclusion of crude glycerin in diets did not affect the chemical composition of the *Longissimus* muscle of Nellore bulls (*P* > 0.10, Table 3).

**Table 3:** Centesimal composition of *Longissimus* muscle of Nellore cattle fed diets containing up to 30% crude glycerin.

Item^1^	Treatments (% crude glycerin)					Contrast, *P*-value^2^			SEM
	G0	G7.5	G15	G22.5	G30	L	Q	0 × Gly	
Moisture	76.3	75.1	76.0	75.2	75.8	NS^3^	NS	NS	1.1
Protein	21.8	22.1	21.2	22.4	21.8	NS	NS	NS	0.7
Fat	2.1	2.5	2.3	2.3	1.9	NS	NS	NS	0.6
Mineral matter	0.9	1.0	0.9	1.0	1.0	NS	NS	NS	0.1

^1^g/100g of meat.

^2^Linear, Quadratic, Control treatment × glycerin treatments.

^3^NS = Not significant,

### Meat and subcutaneous fat color and meat pH

The only color parameter (a*, b* and L*), measured both in meat and subcutaneous fat, which was affected by the inclusion of crude glycerin in cattle diets, was the luminosity index of fat. The L* of fat was greater when glycerin was fed at either 0 or 30% of the diet (64.46 vs. 63.40), however intermediate inclusions of glycerin (7.5, 15 and 22.5%) reduced L* of fat to 61.61, 58.63 and 60.55, respectively (*P*_*Quad*_. < 0.05; Table 4). No treatment effect was observed in terminal pH of meat (Table 4).

**Table 4:** Qualitative characteristics of *Longissimus* muscle and subcutaneous fat from Nellore cattle fed diets containing up to 30% crude glycerin.

Item^2^	Treatments (% crude glycerin)					Contrast, *P*-value^1^*			SEM
	G0	G7.5	G15	G22.5	G30	L	Q	0 × Gly	
L_meat_	33.8	32.6	31.4	31.8	33.4	NS	NS	NS	5.3
A_meat_	13.2	12.5	10.9	12.0	13.0	NS	NS	NS	2.1
B_meat_	2.1	3.1	3.6	3.7	3.7	NS	NS	NS	1.4
L_fat_	64.5	61.6	58.6	60.6	63.4	NS	*	NS	4.8
A_fat_	10.6	9.2	9.7	8.8	9.5	NS	NS	NS	2.2
B_fat_	9.3	8.9	10.6	8.9	10.5	NS	NS	NS	1.7
pH_meat_	5.5	5.5	5.4	5.5	5.6	NS	NS	NS	0.6
WHC, %	74.1	75.5	73.8	75.1	73.8	NS	NS	NS	5.0
SF, N	46.8	46.4	37.4	43.6	39.9	NS	NS	NS	13.0
CL, %	34.9	32.9	31.6	32.0	30.5	NS	NS	NS	5.4
Cholesterol_meat_, mg/g	36.8	30.6	39.9	34.4	27.0	**	*	*	1.7
Cholesterol_fat_, mg/g	105.6	102.6	102.7	108.4	98.7	NS	NS	NS	6.2

^1^Linear (L), Quadratic (Q), Control treatment × glycerin treatments (0 × Gly).

^2^ L_meat_ = luminosity index of meat, A_meat_ = red index of meat, B_meat_ = yellow index of meat, L_fat_ = luminosity index of fat, A_fat_ = red index of fat, B_fat_ = yellow index of fat, WHC = water-holding capacity, SF = Warner-Bratzler shear force, CL = cooking loss.

*P<005,

**P<001, NS = Not significant

### Water holding capacity, cooking loss and Warner–Bratzler shear force

Differences were not observed (*P* > 0.10) for WHC, CL and SF in meat from animals fed crude glycerin (Table 4).

### Meat and subcutaneous fat cholesterol and fatty acid profile

The use of crude glycerin produced slight changes in the fatty acids profile of *Longissimus* muscle and did not affect the fatty acid profile of subcutaneous fat (Tables 5 and 6). A quadratic effect was observed when increasing levels of crude glycerin on the concentration of pentadecanoic acid (C15:0), which was greater for treatment containing 15% of crude glycerin (*P* < 0.01). The same behavior was observed for palmitoleic acid (C16:1cis9, *P* < 0.05). The concentration of the gamma linolenic fatty acid was also affected by increasing the levels of glycerin (*P* < 0.01). Moreover, it was observed a quadratic effect of the inclusion of crude glycerin on concentration of eicosenoic acid in the animal muscle (*P* < 0.05).

**Table 5:** Fatty acid profile of *Longissimus* muscle from Nellore cattle fed diets containing up to 30% crude glycerin.

Item^2^	Treatments (% crude glycerin)					Contrast, *P*-value^1^*			SEM
	G0	G7.5	G15	G22.5	G30	L	Q	0 × Gly	
C10:0	1.1	1.8	0.5	1.4	0.7	NS	NS	NS	0.2
C12:0	1.6	2.4	1.4	1.7	1.3	NS	NS	NS	0.3
C14:0	79.2	105.2	88.2	87.7	60.0	NS	NS	NS	11.9
C14:1c9	22.1	22.6	22.3	13.3	22.0	NS	NS	NS	1.9
C15:0	8.0	13.9	16.4	10.7	6.6	NS	**	NS	0.9
C16:0	592.6	689.2	644.5	679.5	499.1	NS	NS	NS	27.7
C16:1c9	80.0	82.3	81.0	63.7	79.6	NS	*	NS	2.0
C17:0	23.0	38.2	41.1	28.9	18.6	NS	NS	NS	3.6
C17:1	22.1	34.4	36.3	17.6	23.7	NS	NS	NS	6.0
C18:0	292.3	401.6	357.7	473.7	229.5	NS	*	NS	16.2
C18:1n9c	856.9	965.3	926.4	810.9	868.9	NS	NS	NS	28.5
C18:1c11	46.1	48.4	37.9	52.1	36.4	NS	NS	NS	2.0
C18:2n6	40.5	65.5	32.3	47.2	45.7	NS	NS	NS	10.9
C18:3n6	0.9	2.0	2.2	2.7	0.8	NS	**	**	0.1
C18:3n3	2.2	4.0	3.8	2.2	2.4	NS	NS	NS	0.4
C18:2c9t11	5.9	8.0	7.4	6.0	6.0	NS	NS	NS	1.0
C20:0	2.6	3.7	2.4	3.9	3.3	NS	NS	NS	0.4
C20:1n9	4.1	4.1	3.0	2.3	6.6	NS	*	NS	0.5
C20:2	0.6	0.9	0.5	0.7	0.6	NS	NS	NS	0.1
C20:3n6	1.1	2.7	2.3	1.6	1.4	NS	NS	NS	0.7
C20:4n6	1.7	8.2	7.4	0.8	4.8	NS	NS	NS	2.7
C20:5n3	0.6	1.6	1.1	2.1	0.7	NS	NS	NS	0.3
C22:3n3	0.1	0.9	0.6	0.1	0.3	NS	NS	NS	0.2
C24:1n9	1.1	2.8	3.6	0.9	1.9	NS	NS	NS	0.9

^1^mg/100g of meat.

^2^Linear (L), Quadratic (Q), Control treatment × glycerin treatments (0 × Gly).

*P<005,

**P<001, NS = Not significant.

**Table 6:** Fatty acid profile of subcutaneous fat from Nellore cattle fed diets containing up to 30% crude glycerin.

Item^2^	Treatments (% crude glycerin)					Contrast, *P*-value^1^			SEM
	G0	G7.5	G15	G22.5	G30	L	Q	0 × Gly	
C10:0	78	59	79	59	69	NS^3^	NS	NS	10
C12:0	104.2	85.6	115.6	75.6	99.9	NS	NS	NS	9
C14:0	4578	4397	6057	4477	4371	NS	NS	NS	352
C14:1c9	1285	1318	2228	1168	1352	NS	NS	NS	198
C15:0	576	548	518	428	530	NS	NS	NS	94
C16:0	25382	26530	33110	28800	24766	NS	NS	NS	1003
C16:1c9	3300	3328	5288	3058	3597	NS	NS	NS	300
C17:0	1514	1364	713	874	1411	NS	NS	NS	180
C17:1	1140	998	578	448	1127	NS	NS	NS	146
C18:0	14415	14319	11999	16859	14507	NS	NS	NS	1058
C18:1n9c	42948	42498	34128	38638	43620	NS	NS	NS	1581
C18:1c11	2142	2029	2479	2409	1990	NS	NS	NS	170
C18:2n6	1336	1371	1591	1511	1526	NS	NS	NS	185
C18:3n6	74	82	62	82	74	NS	NS	NS	14
C18:3n3	99	112	142	122	98	NS	NS	NS	18
C18:2c9t11	590	520	660	620	420	NS	NS	NS	54
C20:0	133	127	67	168	150	NS	NS	NS	12
C20:1n9	248	288	158	178	225	NS	NS	NS	54
C20:2	23	25	25	25	26	NS	NS	NS	10

^1^mg/100g of meat.

^2^Linear, Quadratic, Control treatment *×* glycerin treatments.

^3^NS = Not significant.

Regarding the enzymatic activity indexes, a quadratic effect was observed of dietary treatments on the enzymes delta-9 desaturase 16 and delta 9 desaturase 18 (*P* < 0.05, Table 7).

**Table 7:** The Δ9 desaturase and elongase enzyme activity indices, and atherogenicity index of *Longissimus* muscle and subcutaneous fat from Nellore cattle fed diets containing up to 30% crude glycerin.

Item	Treatments (% crude glycerin)					Contrast, *P*-value^1^*			SEM
	G0	G7.5	G15	G22.5	G30	L	Q	0 × Gly	
***Longissimus* muscle**									
Δ9 desaturase 16	11.9	10.7	10.7	8.5	13.6	NS	*	NS	0.5
Δ9 desaturase 18	74.4	70.7	71.9	63.6	78.6	NS	*	NS	1.4
Elongase	63.2	63.9	63.5	63.2	65.5	NS	NS	NS	1.4
Atherogenicity	0.9	1.0	0.9	1.1	0.7	NS	NS	NS	0.1
**Subcutaneous fat**									
Δ9 desaturase 16	11.4	11.2	14.1	9.4	12.7	NS	NS	NS	0.9
Δ9 desaturase 18	74.9	74.7	74.5	70.0	74.9	NS	NS	NS	1.7
Elongase	66.6	65.5	54.8	63.5	67.2	NS	NS	NS	1.6
Atherogenicity	0.9	0.9	1.2	1.0	0.8	NS	NS	NS	0.1

^1^Linear (L), Quadratic (Q), Control treatment × glycerin treatments (0 × Gly).

*P<005, NS = Not significant.

*Δ*9 *desaturase* 16 = 100[(*C*16:1*cis*9)/(*C*16:1*cis*9 + *C*16:0)].

*Δ*9 *desaturase* 18 = 100[(*C*18:1*cis*9)/(*C*18:1*cis*9 + *C*18:0)].

*Elongase* = 100[(*C*18:0 + *C*18:1*cis*9)/(*C*16:0 + *C*16:1*cis*9 + *C*18:0 + *C*18:1*cis*9)].

*Atherogenicity* = [*C*12:0 + 4(*C*14:0) + *C*16:0]/*ΣUFA*.

Among total concentration of SFA, MUFA, and polyunsaturated (PUFA) fatty acids in *Longissimus* muscle of animals submitted to different treatments, only MUFA was significantly altered (*P* < 0.05, Table 8). However, the total concentrations of oleic acid, the main monounsaturated fatty acid of *Longissimus* muscle was not significantly affected by the treatments and the trend observed for their concentration was similar to other monounsaturated fatty acids.

**Table 8:** Total saturated fatty acids (SFA), unsaturated fatty acids (UFA), polyunsaturated fatty acids (PUFA), and unsaturated:Saturated ratio (UFA:SFA) of *Longissimus* muscle and subcutaneous fat from Nellore cattle fed diets containing up to 30% crude glycerin.

Item	Treatments (% crude glycerin)					Contrast, *P*-value^1^*			SEM
	G0	G7.5	G15	G22.5	G30	L	Q	0 × Gly	
***Longissimus* muscle**									
SFA, mg/100g	1001	1256	1156	1288	818	NS	NS	NS	44
UFA, mg/100g	1079	1254	1168	1023	1102	NS	NS	NS	44
MUFA, mg/100g	1030	1160	1110	959	1039	NS	*	NS	31
PUFA, mg/100g	49	94	57	63	63	NS	NS	NS	14
UFA:SFA	1.1	1.0	0.9	0.8	1.3	NS	NS	NS	0.2
n-6:n-3	15.0	13.5	9.7	11.2	17.5	NS	NS	NS	1.2
**Subcutaneous fat**									
SFA, mg/100g	46779	47431	52661	51741	45905	NS	NS	NS	1717
UFA, mg/100g	53221	52569	47339	48259	54095	NS	NS	NS	1717
MUFA, mg/100g	51063	50460	44860	45900	51921	NS	NS	NS	1547
PUFA, mg/100g	2158	2110	2480	2360	2174	NS	NS	NS	231
UFA:SFA	1.1	1.2	1.1	1.1	1.0	NS	NS	NS	0.1
n-6:n-3	13.2	14.1	12.2	14.0	16.1	NS	NS	NS	2.4

^1^Linear (L), Quadratic (Q), Control treatment × glycerin treatments (0 × Gly).

*P<005, NS = Not significant.

### Sensory attributes

The trained evaluators identified that the inclusion of crude glycerin increased the score of flavor and greasy intensity of the meat (*P*_*Lin*._ < 0.01, Table 9). It was also observed greater tenderness in the meat of animals fed 15% crude glycerin (*P*_*Quad*._ < 0.05). The top scores for juiciness were awarded to meat from animals fed treatments containing crude glycerin compared to the control diet (0 × Gly, *P* < 0.05), with the highest score given to treatments with 30% crude glycerin.

**Table 9:** Average scores (scale from 0 to 9 points) for sensory attributes of meat from Nellore cattle fed up to 30% crude glycerin.

Item	Treatments (% crude glycerin)					Contrast, *P*-value^1^*			SEM
	G0	G7.5	G15	G22.5	G30	L	Q	0 × Gly	
Appearance	4.4	5.2	4.5	4.5	4.4	NS	NS	NS	1.6
Odor intensity	4.1	5.3	3.2	5.4	3.5	NS	NS	NS	1.6
Flavor intensity	4.8	4.9	5.6	5.6	6.4	**	NS	NS	1.9
Tenderness	3.1	3.6	4.6	2.7	3.3	NS	*	NS	1.5
Juiciness	5.1	4.3	6.6	6.9	6.8	**	NS	*	2.0
Greasy intensity	5.1	4.7	6.3	6.2	7.0	**	NS	NS	1.9
Overall acceptance	4.6	5.9	3.6	6.4	4.2	NS	NS	NS	1.7

^1^Linear (L), Quadratic (Q), Control treatment × glycerin treatments (0 × Gly).

*P<005,

**P<001, NS = Not significant.

## Discussion

### Meat centesimal composition

The analysis of the chemical composition of food aims to gather up to date and reliable information of the final product in order to establish a true nutritional labeling, so consumers really know what they will consume. The proper verification of the nutritional value allows establishing diets and nutritional goals for a better quality of life of humans [26].

In this study, the chemical composition of the *Longissimus* muscle was not altered by the increasing inclusion of crude glycerin in cattle diets. The lack of significant differences among treatments allows us to state that the nutritional quality of meat was maintained without any prejudice for the composition of the final product. The average values of moisture, protein, fat and mineral matter, for all the treatments, were respectively 75.68, 21.85, 23.02 and 0.96%, very similar to those reported by Prado *et al*. [27], Fernandes *et al*. [28] and Françozo *et al*. [29]. Thus, despite the metabolism of glycerol and its conversion to propionate in the rumen, there was not an increased intramuscular fat deposition, which might be expected.

### Meat and subcutaneous fat color and meat pH

The diet is a potential tool used to manipulate the meat quality, such as the color [30]. Therefore, differences in the rate of tissue deposition and use of glycogen can influence the final color. For other color indicators measured, the level of inclusion of crude glycerin in the diets was not enough to promote changes in pH and meat color, similarly reported by others studies, in that color was not influenced by glycerol feeding [13 – 29 – 31 – 32]. On the other hand, Carvalho *et al*. [8] reported that the inclusion of crude glycerin in beef cattle diets (up to 18% of diet`s dry matter) positively affected the meat color. The average values of L*, a*, and b* for meat, found in the present study, are very close to those reported by Muchenje *et al*. [33], who observed values of 33 to 41 for L*, 11.1 to 23.6 for a*, and 6.1 to 11.3 for b*.

The appearance of the meat regarding to color and brightness is an important aspect of quality that influences the attractiveness of meat to consumers at the time of the purchase [34]. Consumers are increasingly demanding for quality and variety of products [35]. This way, the appearance of meat, such as color, has a decisive impact on the choice and consumer acceptance [36 – 37].

### Water holding capacity, cooking loss and Warner–Bratzler shear force

Recent studies have shown that the addition of crude glycerin in diets does not change WHC, CL and SF [13 – 29 – 31]. In ruminants, approximately 80% of glycerol is transformed in the rumen into volatile fatty acids, suggesting a low absorption of the unchanged glycerol molecule, then unchanged cell osmotic pressure, the intracellular water content and the water holding capacity [11]. Furthermore, the feeding used in the production system is an important factor in the tenderness of the meat, since it is involved in the storage of glycogen and modulation of final pH [38]. As the diets had similar energy levels, they had no dietary effect on postmortem glycolysis and the final pH. The obtained SF results with 15%, 22.5% and 30% of crude glycerin used in diet (< 45.1 N) ensured a tenderness that should result in high consumer acceptance [39].

### Meat and subcutaneous fat cholesterol and fatty acid profile

Cholesterol of meat decreased for cattle fed 7.5 and 30% crude glycerin (30.62 vs. 26.97), but treatments G0, G15 and G22.5 increased cholesterol of meat to 36.81, 39.87 and 34.40, respectively (*P*_*Lin*._ < 0.01; P _Quad_.< 0.05; Table 4). However, the cholesterol levels in this study were generally lower than those considered normal for different bovine cuts (58.3–83.4 mg/100 g), according to Werdi Pratiwi *et al*. [40]. It can be inferred that young, non-castrated Zebu cattle fed diets with low ether extract content, reduces meat cholesterol concentration. These animals are likely using cholesterol, during this stage of their development, to produce hormones. Furthermore, the muscle cholesterol concentrations vary, depending on the needs and functions of cell membranes, increasing its solubility when large proportions of saturated fatty acids are present [41 – 42]. Details of cholesterol metabolism in ruminants are not fully elucidated, despite the extensive knowledge in other species [43]. An increased meat deposition of cholesterol can be associated with a lower deposition of C18:0 in muscle. This suggests that C18:0, which is one of the final products of fatty acid synthesis, either desaturated into C18:1 cis-9, or acting as a modulator of cholesterol synthesis [44].

Because high cholesterol consumption by humans may be related to high incidence of cardiovascular diseases and certain types of cancer [45 – 46], cattle fed crude glycerin could be advantageous to consumers wishing to lower their dietary cholesterol intake, while continuing to consume beef.

The pentadecanoic acid is a fatty acid synthesized by ruminal bacteria and found in low concentration in bovine muscle. Vahmani *et al*. [47] evaluated the lipid profile of Canadian beef and the pentadecanoic acid represented about 0.45% of total fatty acids. Whereas the experimental diets had relatively similar fatty acids concentrations, therefore it cannot be concluded that its increase is due to the presence in the diet.

The rumen environment may have become more suitable to microbial growth at 15% of crude glycerin, since greater levels of crude glycerin promoted reductions on the concentration of C15:0, and replacing corn by glycerin may have contributed to this achievement, as the reduction can be considered significant. The diet of the control group, with no crude glycerin contained 35% of ground corn while the diet with 15% crude glycerin, only 18% corn.

Palmitoleic acid is a monounsaturated fatty acid that is produced almost exclusively via desaturation of palmitic acid by the delta-9 desaturase (SCD-1), and the effects of diets on the activity index of the delta-9 desaturase 16 support it. According to Duckett *et al*. [48], there are few dietary sources of this fatty acid, which has been related to insulin sensitivity and intramuscular adipogenesis. Authors had conducted an infusion of C16:1 for 28 days in sheep and verified a reduction in the daily weight gain, the size of intramuscular adipocytes and the total content of lipids. Furthermore, according to them, palmitoleic acid causes changes in the expression of genes that regulate glucose uptake and fatty acid oxidation in muscles. Regarding the stearic acid, a quadratic effect was observed due to the increasing levels of crude glycerin, explained by its effects on the enzyme Δ-9 desaturase 16. Despite the increasing of the concentration of this fatty acid is important in quantitative terms, the stearic acid has no impact on the greatest levels of serum LDL. Around 90% of stearic acid available in the diet can be absorbed in the intestine, and its rapid conversion to oleic acid would prevent the negative effects on the serum concentration of LDL. This rapid conversion does not occur with palmitic acid, because there is no adverse effect on the LDL, once it needs to be lengthened to stearic acid and subsequently desaturated [49].

The gamma linolenic acid (18:3n6), by promoting the activation of the PPAR, can be essential in the transcription of genes involved in lipid and glucose homeostasis [50 – 51]. Moreover, the ingestion of fatty acids through beef during pregnancy is of great importance, because its constraint may result in increased risk for obesity, insulin resistance and elevated serum cholesterol concentrations in adulthood [52].

The concentration of eicosenoic acid (C20:1n9) was altered in present study treatments, therefore it cannot be concluded that the increase in its concentration is a direct effect of the increase in its intake or intestinal absorption. According to Vahmani *et al*. [47], regarding the fatty acids in beef, it appears that eicosenoic acid, in quantitative terms, is the twenty-seventh fatty acid found in greater quantities in a total of eighty-five fatty acids, with an average concentration of 0.21% which is close to the results found in some of the treatments of this research.

The enzymes delta-9 desaturase 16 and delta 9 desaturase 18 can be inhibited by linoleic and linolenic acids, as reported by Daniel *et al*. [53]. In the present study, diets containing increased levels of glycerin also showed increasing levels of linolenic acid, which could explain the reduction in enzyme activities indexes.

Changes observed in MUFA reflect the changes found for palmitoleic and eicosenoic fatty acids.

### Sensory attributes

Sensory analysis is of great importance in controlling and maintaining the quality of end products. The profile of meat with desirable attributes is usually established by the consumer, and involves characteristics measured by sight, touch, smell, taste and hearing.

In this study, the sensory analysis showed that the increasing inclusion of crude glycerin in diets promoted improvement in sensory characteristics of the meat. Increasing scores were assigned by trained evaluators for flavor intensity (Table 9). This may be directly related to juiciness of the meat, attribute that also received notes as increasing inclusion of crude glycerin in diets, with greater scores for treatments G15, G22.5 and G30. However, despite the absence of significant differences among treatments for moisture and fat composition, the trained evaluators had a perception to greasy intensity. This fact may have induced psychologically tasters for a greater perception of tasty and juicy meat, reflecting the final grade of sensory analysis. The greatest tenderness of the meat observed with the inclusion of 15% crude glycerin agrees with the lower numerical value of shear force observed for the same treatment (Table 4).

The lack of effects among the treatments on the a* and b* of *Longissimus* muscle could be explained by the muscle fatty acid profile. The oxidation of oxymyoglobin to metmyoglobin can be accelerated by the products of the lipid oxidation. It is possible that the generation of these products from PUFA had occurred similarly among treatments, as the muscle concentrations of PUFA, which are more susceptible to oxidation, were similar among treatments.

## Conclusions

The meat quality can be changed with the inclusion of high concentrations of crude glycerin in diets for Nellore bulls (up to 30% of total DM), such as the cholesterol content and sensory attributes. This by-product has the potential to change ruminal fermentation, resulting in changes in odd-chain fatty acid concentration in meat, and fatty acids correlated with meat flavor (MUFA) and human health (C18:3n6).

## Supporting information

S1 File Raw data. (XLSX)
